# Does sleep-disordered breathing add to impairments in academic performance and brain structure usually observed in children with overweight/obesity?

**DOI:** 10.1007/s00431-022-04403-0

**Published:** 2022-02-10

**Authors:** Lucia V. Torres-Lopez, Cristina Cadenas-Sanchez, Jairo H. Migueles, Irene Esteban-Cornejo, Pablo Molina-Garcia, Charles H. Hillman, Andres Catena, Francisco B. Ortega

**Affiliations:** 1 PROFITH “PROmoting FITness and Health Through Physical Activity” Research Group, Department of Physical Education and Sports, Faculty of Sport Sciences, Sport and Health University Research Institute (iMUDS), University of Granada, Granada, 18011 Spain; 2 Institute for Innovation and Sustainable Development in the Food Chain (IS-FOOD), Public University of Navarra, Pamplona, Spain; 3 Department of Health, Medicine and Caring Sciences, Linköping University, Linköping, Sweden; 4 Department of Biosciences and Nutrition, Karolinska Institutet, Huddinge, 14183 Sweden; 5 Center for Cognitive and Brain Health, Department of Psychology, Department of Physical Therapy, Movement, and Rehabilitation Sciences, Northeastern University, Boston, MA USA; 6 School of Psychology, University of Granada, Granada, Spain; 7 Faculty of Sport and Health Sciences, University of Jyväskylä, Jyväskylä, Finland

**Keywords:** Preadolescents, Childhood obesity, Obstructive sleep apnea, Academic achievement, Brain health

## Abstract

Approximately 4–11% of children suffer from sleep-disordered breathing (SDB), and children with obesity are at increased risk. Both obesity and SDB have been separately associated with poorer brain health, yet whether SDB severity affects brain health in children with obesity remains unanswered. This study aimed to examine associations of SDB severity with academic performance and brain structure (i.e., total brain and gray and white matter volumes and gray matter volume in the hippocampus) in children with overweight/obesity. One hundred nine children aged 8–12 years with overweight/obesity were included. SDB severity and its subscales (i.e., snoring, daytime sleepiness, and inattention/hyperactivity) were evaluated via the Pediatric Sleep Questionnaire (PSQ), and academic performance was evaluated with the Woodcock-Muñoz standardized test and school grades. Brain structure was assessed by magnetic resonance imaging. SDB severity was not associated with academic performance measured by the standardized test (all |β|> 0.160, *P* > 0.076), yet it was associated with the school grade point average (β = -0.226, *P* = 0.007) and natural and social science grades (β = -0.269, *P* = 0.024). Intention/hyperactivity seemed to drive these associations. No associations were found between SDB severity and the remaining school grades (all β < -0.188, *P* > 0.065) or brain volumes (all *P* > 0.05).

*Conclusion*: Our study shows that SDB severity was associated with lower school grades, yet it was not associated with the standardized measurement of academic performance or with brain volumes in children with overweight/obesity. SDB severity may add to academic problems in children beyond the effects contributed by overweight/obesity status alone.

**Table:** 

**What is Known:** *• Sleep-disordered breathing (SDB) may affect brain structure and academic performance in children.* *• Children with overweight/obesity are at higher risk for the development of SDB, yet the comorbid obesity-SDB relationship with brain health has not been investigated thus far.*	
**What is New:** *• To our knowledge, this is the first study examining the associations of comorbid obesity-SDB severity with brain volumes and academic performance in children.* *• SDB symptoms may adversely affect academic performance at school in children with overweight/obesity, beyond the effects of weight status alone.*

**What is Known:**

*• Sleep-disordered breathing (SDB) may affect brain structure and academic performance in children.*

*• Children with overweight/obesity are at higher risk for the development of SDB, yet the comorbid obesity-SDB relationship with brain health has not been investigated thus far.*

**What is New:**

*• To our knowledge, this is the first study examining the associations of comorbid obesity-SDB severity with brain volumes and academic performance in children.*

*• SDB symptoms may adversely affect academic performance at school in children with overweight/obesity, beyond the effects of weight status alone.*

## Introduction

Pediatric obstructive sleep-disordered breathing (SDB) has been recognized as a common health problem [1]. The European Respiratory Society Task Force defined obstructive SDB as a set of respiratory tract dysfunctions during sleep, which are characterized by snoring and/or increased effort to breathe caused by a narrowing of the pharynx and increased airway resistance [2]. SDB affects approximately 4–11% of children, with a range of severities from primary snoring to obstructive sleep apnea (OSA) [3, 4]. In a previous study including children 6–15 years of age, those with obesity were at higher risk for SDB (20.8%) than their normal-weight peers (6.3%) [4]. Among children with SDB, obesity has been associated with higher SDB severity [5, 6]. These data clearly support the idea that there is an urgent need to address SDB severity in children with overweight/obesity.

A consensus statement by the European Respiratory Society Task Force on the diagnosis and management of SDB in childhood concluded that children with SDB may present an increased prevalence of academic difficulties and cognitive deficits [2]. Relatedly, some previous studies have found that children with SDB might present impaired academic [7–10] and neurocognitive [11, 12] performance and that SDB in children as young as 3 years old predicted significantly poorer performance on oral language assessments at 8 years old [13]. However, these findings have been inconsistent [11]. Specifically, research in children and adolescents with overweight/obesity and comorbid SDB has shown lower neurocognitive performance [14, 15] and contradictory findings on academic performance [16–19]. Thus, children with overweight/obesity may be at increased risk of neurocognitive deficits when they present with SDB symptoms [14, 15, 20]. However, more research is needed to better understand the associations with academic performance.

Childhood is an important period for the development and maturation of the brain [21, 22], and the hippocampus is a key subcortical structure involved in memory consolidation during wakefulness and sleep [23, 24]. Among the studies investigating pediatric SDB-brain structure links [25–32], only a few found significant associations in the hippocampus [29, 31, 33]. Beyond the hippocampus, a recent review concluded that obstructive SDB in children was associated with magnetic resonance imaging (MRI)-assessed brain structure [34]. For example, children with OSA presented smaller regional gray matter volume (GMV) in areas related to cognitive control (i.e., frontal and prefrontal cortices, parietal cortices, temporal lobes, and brainstem) than their peers without any sleep disorder [32]. Conversely, other studies have shown no clear evidence of an association between OSA and brain structure [35, 36]. Regarding children with obesity, they have been shown to more prone to suffer from OSA than children with normal weight [37], and they often present impaired regional gray and white matter development [38]. It remains to be elucidated whether SDB increases brain structure impairments in children with overweight/obesity.

Evidence is lacking regarding the joint role of SDB and obesity on academic performance and brain structure in children. Identifying the associations of SDB severity with academic performance and brain structure in the context of childhood obesity could provide meaningful information for public health systems to prevent or attenuate the health-related consequences of obesity with efficient interventions. Therefore, the present study investigated the associations of SDB severity with academic performance using two different measures (i.e., standardized tests and school grades) and with brain structure (i.e., total brain volumes and hippocampal volume as a primary region of interest) in children with overweight/obesity. Identifying these associations might help to provide a better understanding of the influencing role of SDB on brain health beyond weight status alone.

## Methods

### Study design and participants

We used baseline data from ActiveBrains, a randomized controlled trial intended to investigate the effects of an exercise program on the brain and cognition in children with overweight/obesity. Data were collected from November 2014 to February 2016 in a total of 110 overweight/obese children from Granada (Spain). Of them, one child was excluded for being a nonnative Spanish speaker and did not reach the required threshold in Spanish reading comprehension to be properly evaluated. Thus, a total of 109 children (10.04 ± 1.12 years old; 45 girls) were included in this cross-sectional study. Most of the participants were European Caucasian (95%), with only ~ 5% reported being of other ethnicities. A detailed description of the study protocol, aims, and methods has been published elsewhere [39]. All participants met the following inclusion criteria: (1) overweight or obesity, based on the sex- and age-specific international body mass index cutoff points proposed by the World Obesity Federation [40, 41]; (2) age between 8 and 12 years old; (3) no physical disabilities or neurological disorders that could affect physical performance; (4) prepubertal status according to the Tanner stages [42]; and (5) for the girls, no menarche.

Legal guardians were informed of the purpose of the study, and written informed consent for all participants was obtained. This study was conducted in accordance with the Declaration of Helsinki. The study protocol was approved by the Ethics Committee on Human Research (CEIH) of the University of Granada and registered at ClinicalTrials.gov (identifier: NCT02295072).

### Procedures and measurements

#### Sleep-disordered breathing

The children’s legal guardians completed the Spanish version of the Pediatric Sleep Questionnaire (PSQ) to assess SDB, which has shown high reliability and internal consistency [43]. This questionnaire has been validated for the identification of SDB [44, 45]. A sleep-related breathing disorders (SRBD) scale (scores could range from 0 to 1) was calculated from the 22 items of the questionnaire, with higher scores indicating greater severity [45]. The SRBD scale consisted of 22 closed response question items as a reduced version of the PSQ, which were divided into the following three subscales [44, 45]: snoring (four items), sleepiness (four items), and inattention/hyperactivity (six items). These subscales are valid and reliable for assessing SDB in children [44]. The answer options for each item included “yes,” “no,” and “I do not know.” In the last domain of the questionnaire (inattention/hyperactivity subscale), the answers were structured as “never,” “sometimes,” “often,” or “almost always.” To be consistent with these answer options throughout the questionnaire, one of the sections was recategorized as follows: “never” and “sometimes” answers were categorized as “no,” whereas “often” and “almost always” answers were categorized as “yes.” The overall scale was calculated as the sum of the affirmative answers divided by 22. Occasional missing answers or responses of “I do not know” were discounted from the denominator when calculating these proportions [46]. Scores > 0.33 were considered suggestive of high risk for pediatric SDB [44, 45]. A recent meta-analysis indicated that the SRBD scale was accurate for detecting OSA in children [47]. The SRBD scale score has been validated as a continuous indicator of SDB severity [6].

#### Academic performance

Academic performance was assessed via the Spanish adaptation of the Woodcock-Johnson III battery (i.e., Woodcock-Muñoz standardized test), which has shown high reliability and is a well-validated measure of academic performance for individuals aged 5–95 years [48]. For this study, we used the standard battery, which contained three tests of mathematics, three tests of reading, and three tests of written language. The assessment was individually conducted for each child by a trained evaluator in a session lasting 100–120 min. The data registered for each child were individually checked by two trained evaluators. All data were processed using Compuscore and Profile software version 3.1. (Riverside Publishing Company, Itasca, IL, USA) and grouped into components (i.e., total achievement, reading, writing, written expression, mathematics, calculation, and calculation skills). For the current study, a standard T-score based on an average of 100 and a standard deviation of 15 was obtained for the following academic performance indicators: mathematics, reading, writing, and total achievement. Academic performance tests and MRI were conducted within 1 to 3 weeks of each other.

As a second measure of academic performance, official final school grades, closer to the baseline assessment (± 1.5 months), were used for the analyses in 78% of the study sample (*n* = 85). Data were not missing at random (*n* = 24). The school grades were initially collected in the second wave of participants, so they were missing for the first wave. Since school records were provided in a qualitative manner (i.e., insufficient, sufficient, good, very good, and outstanding) by teachers, we registered the grades based on a scale from 1 to 5. The mean grades in mathematics, Spanish language, English language, and natural and social sciences were recorded. We also determined the individual’s grade point average as the mean score across all subjects.

#### Brain structure

Brain volume was assessed using magnetic resonance imaging (Siemens Magnetom Tim Trio, 3 T, Siemens, Erlangen, Germany) equipped with a 32-channel head coil. High-resolution, T1-weighted images were acquired using a 3D MPRAGE (magnetization-prepared rapid gradient-echo) protocol. Acquisition parameters were as follows: repetition time = 2,300 ms, echo time = 3.1 ms, inversion time = 900 ms, flip angle = 9°, field of view = 256 × 256, acquisition matrix = 320 × 320, 208 slices, and resolution = 0.8 × 0.8 × 0.8 mm. The total acquisition time for the T1 sequence was 7 min and 31 s for each child. Imaging preprocessing included quality control, motion correction, spatial normalization to an MNI (Montreal Neurological Institute) template, and spatial smoothing. Detailed information about preprocessing has been described previously [49]. Some missing data were found for brain volumes (*n* = 9) and hippocampal GMV (*n* = 7). Total brain volume, total white matter volume (WMV), total GMV, and total right and left hippocampal GMV were derived from FreeSurfer software version 5.3.0 (Laboratory for Computational Neuroimaging, Athinoula A. Martinos Center for Biomedical Imaging, Harvard Medical School, Boston MA, USA).

#### Covariates

Age, sex, maternal and paternal education levels, wave of participation (i.e., ActiveBrains was conducted in three waves for logistic reasons, including seasonality and differences between participants from different waves that may affect the associations), and peak height velocity (PHV) were considered potential confounders. Maternal and paternal maximum education levels were obtained by a self-report questionnaire. The responses of the mothers and fathers were classified as primary school, secondary school, and university degree completed. The wave of participation (1, 2, or 3) was categorized into two dichotomous dummy variables. PHV is an indicator of maturity during childhood and adolescence calculated from standing and seated height using Moore’s equations [50].

### Statistical analysis

Descriptive characteristics are presented as the means and standard deviations for continuous variables and as frequencies and percentages for categorical variables. Sex differences were examined by *t* tests and chi-square tests. All variables were checked for normality of distribution using the Kolmogorov–Smirnov test and visual inspection of histograms. After checking for a normal distribution, the SRBD scale and subscales were normalized using Blom’s formula [51] because the distributions were skewed.

Hierarchical linear regression analyses were performed to examine the associations of SDB severity with academic performance and brain structure indicators (i.e., total GMV and WMV, total brain volume, and right and left hippocampal GMV). The stepwise method was used to investigate the influence of potential confounders in the models in step 1 (i.e., age, sex, maternal education level, paternal education level, wave of participation, and PHV). Next, hierarchical regressions were carried out, entering the SRBD scale scores as a predictor in step 2 and each academic performance and brain structure variable as outcomes in separate regression analyses after the inclusion of confounders previously defined in step 1. The same procedure was followed to assess the association of each subscale score from the PSQ (i.e., snoring, daytime sleepiness, inattention/hyperactivity) with academic achievement and brain structure.

The Benjamini–Hochberg procedure was applied to account for random effects in multiple comparisons for each dependent domain (i.e., academic performance assessed by standardized test, academic performance assessed by school grades, brain volumes, and hippocampal GMV) with *q* = 0.1 [52]. All analyses were performed using the Statistical Package for Social Science (IBM SPSS Statistics for Windows, version 22.0, Armonk, NY). The level of significance was set at *p* < 0.05.

## Results

Descriptive characteristics for all participants are presented in Table 1. The results were not reported separately by sex since no significant sex interaction was observed in the models (sex was included as a confounder). Hierarchical linear regression analyses for the association of SDB severity with academic performance and brain structure variables are presented in Table 2. Regarding academic performance measured by standardized testing, SDB severity was not significantly associated with mathematics, reading, writing, or total academic performance (all |β|> 0.160, *P* ≥ 0.076). Regarding school grades, SDB severity was significantly associated with natural and social sciences grades and grade point average (β = -0.226, *P* = 0.024; β = -0.269, *P* = 0.007, respectively). Nonsignificant associations were found between SDB severity and mathematics, Spanish language and English language (all β < -0.188, *P* > 0.065), and SDB severity was not associated with any of the brain outcome measures studied (all *P* > 0.05). Sensitivity analyses were performed by adding BMI as a confounder to previous models, and all associations presented in this study remained unchanged (data not shown). Additionally, hierarchical linear regression analyses were performed to assess potential associations of SDB subscale scores (snoring, sleepiness and inattention/hyperactivity) with academic performance and brain structure, as shown in Table 3. The PSQ inattention/hyperactivity subscale was the only subscale associated with school grades (specifically with mathematics grades, natural and social science grades, and grade point average; all *P* < 0.014). No significant differences were observed in the remaining aspects of academic performance and brain structure variables examined.

**Table 1:** Descriptive characteristics of the participants

		**All**		**Boys**		**Girls**	***P***
	N	Mean ± SD	N	Mean ± SD	N	Mean ± SD	
Age (years)	109	10.04 ± 1.12	64	10.16 ± 1.15	45	9.88 ± 1.08	0.197
Weight (kg)	109	56.21 ± 11.23	64	57.11 ± 11.2	45	54.94 ± 11.28	0.323
Height (cm)	109	144.22 ± 8.41	64	144.98 ± 7.97	45	143.13 ± 8.99	0.261
BMI categories ^a^	109						0.606
Overweight, *n* (%)		28 (25.7)		16 (25)		12 (26.7)	
Obesity class I, *n* (%)		47 (43.1)		30 (46.9)		17 (37.8)	
Obesity class II, *n* (%)		34 (31.2)		18 (28.1)		16 (35.6)	
BMI z-scores ^b^	107	3.04 ± 0.89	63	3.22 ± 1.00	44	2.80 ± 0.63	**0.008**
*Sleep-related breathing disorders (SRBD)*							
SRBD scale (range: 0 to 1)	109	0.19 ± 0.13	64	0.19 ± 0.13	45	0.18 ± 0.13	0.621
SRBD presence, *n* (%)		17(15.6)		10 (15.6)		7 (15.6)	0.992
*Academic performance (Woodcock-Muñoz) *^*c*^							
Mathematics	109	102.06 ± 10.91	65	102.66 ± 11.72	44	101.18 ± 9.64	0.490
Reading	109	108.46 ± 12.68	65	108.83 ± 11.02	44	107.91 ± 14.91	0.711
Writing	109	114.1 ± 12.84	65	112.98 ± 12.13	44	115.75 ± 13.8	0.272
Total achievement	109	109.56 ± 11.83	65	109.51 ± 11.07	44	109.64 ± 13.01	0.956
*Academic performance (school grades)* range: 0 to 5							
Mathematics	85	3.69 ± 1.01	51	3.73 ± 1.01	34	3.63 ± 1.01	0.642
Spanish language	85	3.72 ± 0.97	51	3.61 ± 0.93	34	3.88 ± 1.03	0.211
English language	85	3.65 ± 1.12	51	3.51 ± 1.14	34	3.86 ± 1.08	0.157
Natural and social science	85	3.71 ± 1.02	51	3.64 ± 1.11	34	3.81 ± 0.87	0.424
Grade point average	85	3.77 ± 0.79	51	3.69 ± 0.82	34	3.9 ± 0.73	0.218
*Brain volumes* (mm^3^)							
Total gray matter	100	793.48 ± 66.22	60	819.49 ± 56.13	40	754.48 ± 61.36	** < 0.001**
Total white matter	100	406.81 ± 47.97	60	426.88 ± 42.86	40	376.7 ± 38.9	** < 0.001**
Total brain volume	100	1200.29 ± 106.69	60	1246.37 ± 88.88	40	1131.18 ± 93.71	** < 0.001**
*Hippocampal GMV*							
Right hippocampus	102	3582.42 ± 390.34	60	3675.44 ± 386.45	42	3449.54 ± 360.11	**0.004**
Left hippocampus	102	3454.5 ± 379.77	60	3522.31 ± 387.57	42	3357.62 ± 350.42	**0.030**
Total hippocampus	102	7036.92 ± 694.08	60	7197.75 ± 666.26	42	6807.16 ± 675.3	**0.005**

Data shown are mean ± standard deviation or *n*, %. Differences between sexes were examined by *t*-test or chi-square test. Statistically significant values (*P* < 0.05) are shown in bold. Notes: *SD* standard deviation. ^a^BMI categories (overweight, obesity class I and obesity class II) were based on the sex- and age-specific international BMI cut-points proposed by the World Obesity Federation (https://www.euro.who.int/en/health-topics/disease-prevention/nutrition/a-healthy-lifestyle/body-mass-index-bmi). ^b^BMI z-score based on World Health Organization. ^c^Academic performance indicators were calculated based on standardized scores centered at 100. Standardized composite scores are presented. *BMI* body mass index, *SDB* sleep-disordered breathing, *SRBD* sleep-related breathing disorders, *GMV* gray matter volume

**Table 2:** Hierarchical linear regression for the associations of SDB severity with academic performance, brain volumes, and hippocampal gray matter volume variables

	**SRBD scale**			
	**β**	***R***^**2**^	**CI**	***P***
*Academic performance (Woodcock-Muñoz)*				
Mathematics†	0.023	0.228	(-0.150, 0.196)	0.793
Reading‡	-0.030	0.253	(-0.198, 0.139)	0.729
Writing‡	-0.160	0.177	(-0.338, 0.017)	0.076
Total achievement†	-0.058	0.297	(-0.223, 0.107)	0.488
*Academic performance (school grades)*				
Mathematics‡	-0.176	0.183	(-0.383, 0.026)	0.086
Spanish language‡	-0.188	0.190	(-0.395, 0.012)	0.065
English‡	-0.168	0.149	(-0.379, 0.038)	0.107
Natural and social science†	-0.226	0.250	(-0.427, -0.031)	**0.024**
Grade point average‡	-0.269	0.238	(-0.470, -0.076)	**0.007**
*Brain volumes*				
Total gray matter††	0.058	0.329	(-0.112, 0.225)	0.505
Total white matter‡‡	-0.058	0.450	(-0.212, 0.097)	0.462
Total brain volume††	0.018	0.373	(-0.145, 0.181)	0.828
*Hippocampal GMV*				
Right hippocampus†††	-0.090	0.127	(-0.277, 0.098)	0.345
Left hippocampus‡‡‡	0.004	0.046	(-0.191, 0.199)	0.969
Total hippocampus‡‡‡	-0.043	0.079	(-0.235, 0.148)	0.655

The predictor variable was introduced in separate hierarchical regression models. Potential confounders (i.e., age, sex, maternal education level, paternal education level, wave, and peak height velocity) were included into step 1 of the stepwise regression to test their association to the outcomes so that for each outcome, only the relevant confounders were retained in the final models using the method STEPWISE. In step 2, the exposure variable of interest, SRBD scale was entered using the method ENTER to force to be in the model. β values are standardized coefficients. Bolded font indicates that the specific association surpassed the Benjamini–Hochberg correction for multiple comparisons test (performed for each domain, i.e., academic performance assessed by Woodcock-Muñoz battery, academic performance assessed by school grades, brain volumes, and hippocampal GMV). †Adjusted by age and maternal education level. ‡Adjusted by maternal education level. ††Adjusted by sex and paternal education level. ‡‡Adjusted by age, sex, paternal education level, and peak height velocity. †††Adjusted by sex and peak height velocity. ‡‡‡Adjusted by sex. *SDB* sleep-disordered breathing, *SRBD* sleep-related breathing disorders, *GMV* gray matter volume

**Table 3:** Hierarchical linear regression for the associations of SDB subscales (snoring, sleepiness, and inattention/hyperactivity) with academic performance, brain volumes, and hippocampal gray matter volume variables

	**Snoring**		**Daytime sleepiness**		**Inattention/hyperactivity**	
	**β**	***P***	**β**	***P***	**β**	***P***
*Academic performance (Woodcock-Muñoz)*						
Mathematics†	0.060	0.493	-0.086	0.326	0.028	0.747
Reading‡	0.027	0.751	-0.124	0.146	-0.025	0.768
Writing‡	-0.032	0.720	-0.168	0.059	-0.165	0.070
Total achievement†	0.029	0.726	-0.154	00.063	-0.056	0.506
*Academic performance (school grades)*						
Mathematics‡	0.075	0.461	-0.138	0.181	-0.257	**0.014**
Spanish language‡	0.015	0.886	-0.094	0.365	-0.182	0.083
English‡	0.036	0.731	-0.105	0.320	-0.145	0.180
Natural and social science†	0.029	0.771	-0.204	0.043	-0.253	**0.013**
Grade point average‡	-0.004	0.970	-0.141	0.171	-0.297	**0.004**
*Brain volumes*						
Total gray matter††	-0.007	0.935	-0.006	0.945	0.132	0.130
Total white matter‡‡	0.035	0.657	-0.087	0.267	0.020	0.800
Total brain volume††	0.026	0.749	-0.046	0.579	0.102	0.225
*Hippocampal GMV*						
Right hippocampus†††	-0.133	0.164	-0.020	0.833	-0.090	0.349
Left hippocampus‡‡‡	-0.071	0.471	-0.113	0.255	0.026	0.791
Total hippocampus‡‡‡	-0.096	0.320	-0.080	0.415	-0.033	0.735

The predictor variable was introduced in separate hierarchical regression models. Potential confounders (i.e., age, sex, maternal education level, paternal education level, wave, and peak height velocity) were included into step 1 of the stepwise regression to test their association to the outcomes so that for each outcome, only the relevant confounders were retained in the final models using the method STEPWISE. In step 2, the exposure variable of interest (e.g., snoring or sleepiness, or inattention/hyperactivity subscale) was entered using the method ENTER to force to be in the model. β values are standardized coefficients. Bolded font indicates that the specific association surpassed the Benjamini–Hochberg correction for multiple comparisons test (performed for each domain, i.e., academic performance assessed by Woodcock-Muñoz battery, academic performance assessed by school grades, brain volumes, and hippocampal GMV). †Adjusted by age and maternal education level. ‡Adjusted by maternal education level. ††Adjusted by sex and paternal education level. ‡‡Adjusted by age, sex, paternal education level and peak height velocity. †††Adjusted by sex and peak height velocity. ‡‡‡Adjusted by sex. *SDB* sleep-disordered breathing, *GMV* gray matter volume

## Discussion

The main findings of this study support the ideas that (1) SDB severity was related to lower academic performance at school, particularly with natural and social science grades and grade point average, but not with academic performance measured by a standardized test; (2) inattention/hyperactivity seemed to drive these associations between SDB severity and school grades; and (3) SDB severity was not associated with differences in brain structure in children with overweight/obesity. Therefore, this study adds to the literature by showing that SDB symptoms may worsen academic performance in children beyond the effects of weight status alone.

Our study findings suggest a lower academic performance in children with a high risk of SDB than in their peers with a low risk of SDB. It may be that a single assessment through the administration of a standardized test might not fully capture the performance of children across time in the same manner as school grades do. Other possible factors explaining the differences between the school grade and standardized test outcomes may be the different grading systems between schools or the subjective influence of teachers [53]. To the best of our knowledge, only a few studies have focused on children with overweight/obesity with comorbid SDB and academic performance. Collectively, the conclusions across studies have been inconsistent [16–19]. In line with our findings, a previous study showed that academic grades were impaired in children with overweight and SDB compared to children with overweight but without SDB based on reports from parents and children [18]. Beebe et al. [18] found that these associations might be explained by behavioral factors rather than cognitive factors, which may explain why we observed associations with school grades but not with a standardized test. However, other studies measuring other dimensions of school functioning did not observe an impairment in children with SDB compared to children without SDB [19]. This inconsistency might be primarily explained by the different constructs that were measured, since Biggs et al. [19] focused on school functioning as a measure related to quality of life, yet they did not collect school grades. Moreover, recent studies found that overweight/obesity was inversely associated with school performance in childhood [54, 55]. Indeed, both SDB and obesity may adversely influence cognitive functioning [20] and, subsequently, academic performance. Children with overweight/obesity and SDB were at higher risk of neurocognitive deficits than normal-weight children (with and without SDB) [14, 15]. As an alternative explanation, they suggested that obesity might worsen neurocognitive performance in children rather than SDB alone, yet they did not include a non-SDB overweight/obesity group. Hence, we added to the literature by showing that SDB symptoms may worsen already-impaired academic achievement in children with overweight/obesity. However, evidence that affirms the association of obesity with academic performance is not strong enough [56], and future research with a healthy-weight control group and proper sample size should further investigate the interactions between obesity and SDB in association with academic performance.

Taken together, our findings and the abovementioned studies suggested that the previously observed association between SDB and school grades in normal-weight children [7–10, 57, 58] is also apparent in children with overweight or obesity. The mechanisms driving the association between SDB and school performance remain unclear [18]. SDB may produce intermittent hypoxia, hypercapnia, and reoxygenation, along with changes in cerebral blood flow and sleep fragmentation [59], potentially affecting cognitive functioning. Although variability in cognitive function in children with SDB may be explained by a reduction in cerebral oxygenation [60], the pathophysiology of the involvement of the central nervous system during OSA is still debated [61]. However, a recent review found that cerebral oxygenation levels were maintained in this population [34] and were not related to cognitive deficits, but they were associated with behavioral problems in children with SDB [62]. This conclusion may explain the association in our study between SDB and school grades, where children’s behavior, general functioning, and teachers’ influence on children’s behaviors are relevant. Future studies exploring SDB severity with polysomnography and a proper sample size should corroborate or contrast with our findings.

With regard to brain structure, SDB severity was not related to total brain volume or specifically to hippocampal volume in our sample of children with overweight/obesity. Similarly, a study in 10-year-old children found no significant difference between the OSA and control children in total and regional brain volume and density (i.e., GMV, gray/white matter ratio, total brain volume, and gray/total brain ratio) [35]. Unlike our study, a recent review identified that SDB was associated with changes in the brain using MRI in children with normal weight [34]. Likewise, using polysomnography, recent studies determined that normal-weight children (7–11 years) with OSA had smaller regional GMV [32] and WMV [63] than children without OSA. Furthermore, Walter and Horne [33] concluded in a review that previous MRI studies found significant structural changes (i.e., reduced white and gray matter of particular brain areas, including the hippocampus) in children with SDB. Our results may differ from these findings due to the method of measuring SDB (i.e., overnight polysomnography vs. PSQ). It is important to consider that SDB severity in our participants may be mild (i.e., the SRBD average score was 0.19 [SD = 0.13] on a scale with a range from 0 to 1, with 0.33 indicating higher risk for SDB). Other studies in children with higher SDB severity have found regional gray matter deficits [35]. The lower SDB severity in our participants could explain the lack of apparent brain volume deficits and the small-sized associations with academic achievement. The lack of significant associations may be due to the low statistical power reached in this analysis. Only 17 participants presented SDB in this study (15.6%). As such, SDB severity might not have been sufficient to affect myelination and neuronal connectivity in our participants [64]. However, it is difficult to compare our findings to other cohorts since no previous study was conducted with children with overweight/obesity. In adults with OSA, lower GMV in the left hippocampus [65] and in the right hippocampus [66, 67] has been found, although this association was not observed here in the context of childhood obesity. Differing developmental stages [68] and not using polysomnography in our study may have partly explained the lack of observed associations. Thus, larger studies including both normal-weight children and children with overweight/obesity are needed to confirm these findings.

The high prevalence of pediatric obesity worldwide and its associated health-related problems, such as SDB, provide an impetus for the investigation of brain health factors associated with this disorder. Previous research has shown links between SDB and academic performance and brain structure in the pediatric population. However, information on how SDB severity in children with overweight/obesity is associated with academic performance and brain health is limited. Our study contributes to the current knowledge by suggesting that SDB may worsen academic performance in children with overweight/obesity (who often show impaired performance). We also provided a broader picture of academic performance by including two different assessments: school grades and standardized test results. Although we did not find associations with brain structures, it remains to be elucidated whether these changes in school grades have any clinical significance. Some limitations need to be acknowledged. First, this was a cross-sectional study; hence, it does not allow for causal inference for any of the observed associations. Second, our data were obtained from a parent-reported questionnaire instead of polysomnographic data (i.e., the gold standard for the detection of SDB and its severity); however, this questionnaire is valid and reliable for the diagnosis of SDB symptoms. Third, including a group of children with normal weight could be informative toward understanding the interaction between obesity and SDB. Unfortunately, we did not collect data in children with normal weight, and we encourage future researchers to gain insight into this interaction by assessing children with varied weight status. Fourth, teachers may have graded the children from different schools differently. Fifth, our sample size and the prevalence of SDB may have limited the statistical power of the analysis. Future research should confirm our findings. Caution is advised with the extrapolation of these findings to younger children or to other determinants of SDB that were not considered in this study (e.g., adenotonsillar disease, ethnicity, and levels of deprivation). On the other hand, the strengths of this study include the use of MRI for the quantification of GMV in the hippocampus and total brain volume; the focus on children with overweight/obesity, who are particularly vulnerable to sleep disorders; and a comprehensive academic performance assessment using two different methods, i.e., standardized tests and school grades.

## Conclusion

Our study contributes to the literature by indicating that SDB severity was related to lower academic performance at school in children with overweight/obesity, which was driven by inattention/hyperactivity. However, we did not observe associations with academic performance measured by a standardized test or brain structure in children with overweight/obesity. Accordingly, our findings suggest that SDB symptoms may adversely affect learning in school in children with overweight/obesity beyond the effects of weight status alone.

## Data Availability

Not applicable.

## Abbreviations

GMV: Gray matter volume
OSA: Obstructive sleep apnea
SDB: Sleep-disordered breathing
SRBD: Sleep-related breathing disorders
WMV: White matter volume
